# Qualitative Study of Mental Health Improvements with Traditional Cultural Healers in North America

**DOI:** 10.1192/j.eurpsy.2022.277

**Published:** 2022-09-01

**Authors:** L. Mehl-Madrona, B. Mainguy

**Affiliations:** 1 University of Maine, Native Studies/intermedia, Orono, United States of America; 2 Coyote Institute, Education, Ottawa, Canada

**Keywords:** anomalous outcomes, two-eyed seeing, indigenous philosophy, Indigenous people

## Abstract

**Introduction:**

Traditional cultural healers -- their methods and their results -- are often invisible to conventional medical practitioners. When confronted with a result that does not make sense, we often ignore it.

**Objectives:**

We wanted to understand the process that happened between people and traditional cultural healers when these people experienced substantial improvement in mental health without psychiatric treatment.

**Methods:**

We collected 56 case stories from people who consulted traditional cultural healers instead of conventional medical practitioners for serious mental health problems. We confirmed the stories with family members and interviewed the healers as well. We used constructivist grounded theory to explore commonalities in the stories from the clients’ points of view and from the healers’ perspectives. The context is indigenous people in North America.

**Results:**

Patients had a range of diagnoses, including psychotic disorders (12), bipolar disorder (28), and evere unipolar depression (16). Co-morbid anxiety disorders were common (22). Improvement in mental health was associated with participation in ceremonies within a community, building relationships with members of that community, engaging in prescribed daily practices endorsed by that community, with a resulting report of feeling increases in social and spiritual connectedness. The healers believed strongly that the embeddedness in community contributed to the results and that spirit beings played important roles in helping people feel better. The use of psychiatric medications was minimal.

**Conclusions:**

Psychiatry can acknowledge that people can have substantial improvements in mental health when working with traditional cultural healers outside of conventional settings. Studying these settings and results can improve conventional practice.

**Disclosure:**

No significant relationships.

